# A genome-scale RNAi screen for genetic interactors of the dynein co-factor *nud-2* in *Caenorhabditis elegans*

**DOI:** 10.1038/sdata.2018.47

**Published:** 2018-03-20

**Authors:** Helder Rocha, André F. Maia, Reto Gassmann

**Affiliations:** 1 Instituto de Investigação e Inovação em Saúde (i3S), Universidade do Porto, Porto, Portugal; 2 Instituto de Biologia Molecular e Celular (IBMC), Universidade do Porto, Portugal

**Keywords:** RNAi, Dynein, Genetic interaction

## Abstract

Cytoplasmic dynein 1 (dynein) is the predominant microtubule minus end-directed motor in animals and participates in a wide range of cellular processes, including membrane trafficking, nuclear migration, and cell division. Dynein's functional diversity depends on co-factors that regulate its subcellular localization, interaction with cargo, and motor activity. The ubiquitous co-factor nuclear distribution gene E (NudE) is implicated in many of dynein's functions, and mutations in NudE cause the brain developmental disease microcephaly. To identify genetic interactors of the *Caenorhabditis elegans* NudE homolog *nud-2*, we performed a genome-wide RNAi screen with the null allele *nud-2(ok949)*, which compromises dynein function but leaves animals viable and fertile. Using bacterial feeding to deliver dsRNAs in a 96-well liquid format and a semi-automated fluorescence microscopy approach for counting parents and progeny, we screened 19762 bacterial clones and identified 38 genes whose inhibition caused enhanced lethality in *nud-2(ok949)* relative to the *nud-2(+)* control. Further study of these genes, many of which participate in cell division, promises to provide insight into the function and regulation of dynein.

## Background & Summary

Animal cells rely on molecular motor proteins to generate forces and transport cargo along cytoskeletal tracks. Microtubule-based transport is critical for a wide range of cellular and developmental processes, and its dysfunction is recognized as an important contributor to neurological disease^1^. The mega-dalton motor complex cytoplasmic dynein 1 (dynein) is involved in virtually all processes that require microtubule minus end–directed motility, including intracellular trafficking, organelle positioning, and the organization of microtubule arrays^2^. During cell division, dynein is implicated in chromosome movement, assembly and positioning of the bipolar spindle, and the control of spindle assembly checkpoint signalling^3^. Dynein's functional diversity is facilitated by co-factors and cargo-specific adaptor proteins that regulate localization and activity of the motor^4,5^. For many dynein-dependent processes, the inventory of participating adaptors and regulators is likely incomplete.

**Figure 1:**
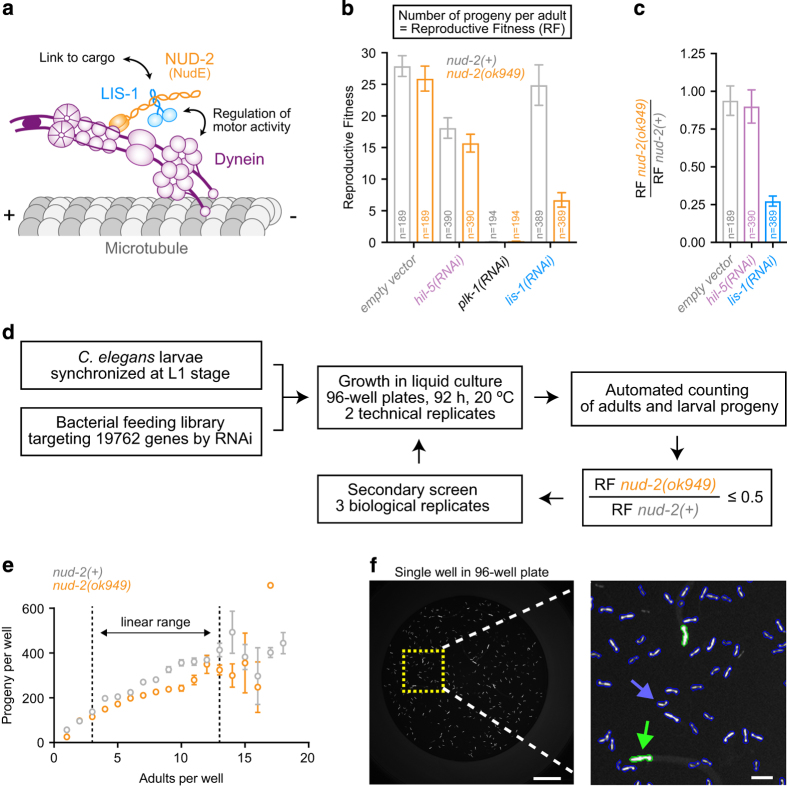
Screening strategy. (**a**) Cartoon summarizing the two main functions of the dynein co-factors NudE and Lis1: regulation of motor activity and recruitment of dynein to cargo. (**b**) Proof of concept experiments. Graph shows the number of larval progeny per adult, termed reproductive fitness (RF), in the *nud-2(+)* control and the null allele *nud-2(ok949)*, after targeting genes with dsRNA using the bacterial feeding method in a 96-well liquid format. Empty dsRNA vector and *hil-5(RNAi)* are negative controls, *plk-1(RNAi)* is a control for the efficiency of RNAi, and *lis-1(RNAi)* is the positive control that results in enhanced lethality in *nud-2(ok949)* relative to *nud-2(+)*. Error bars represent the SEM with a 95 % confidence interval, and *n* denotes the number of adults whose progeny were counted. (**c**) The RF ratio, defined as the RF of *nud-2(ok949)* divided by the RF of *nud-2(+)* for the conditions shown in *(b)*. Error bars represent the SEM with a 95 % confidence interval. (**d**) Flowchart of the screening protocol. The primary screen was performed in two technical replicates per dsRNA vector and strain. The secondary screen was performed in three biological replicates with two technical replicates each. An RF ratio of 0.5 was used as a cutoff for genetic interactors of *nud-2*. (**e**) The number of progeny per well plotted against the number of parents per well for the primary genome-wide screen. For 3 - 13 parents treated with empty dsRNA vector the correlation is linear and thus suitable for evaluation of reproductive fitness. Error bars represent the SEM. (**f**) Example image of a screening well captured with a 2x objective. Blow up on the right shows GFP-labelled pharynxes, which can be automatically assigned to parents (green) or progeny (blue) based on their size. Scale bar, 1 mm; blow-up, 200 μm.

**Figure 2:**
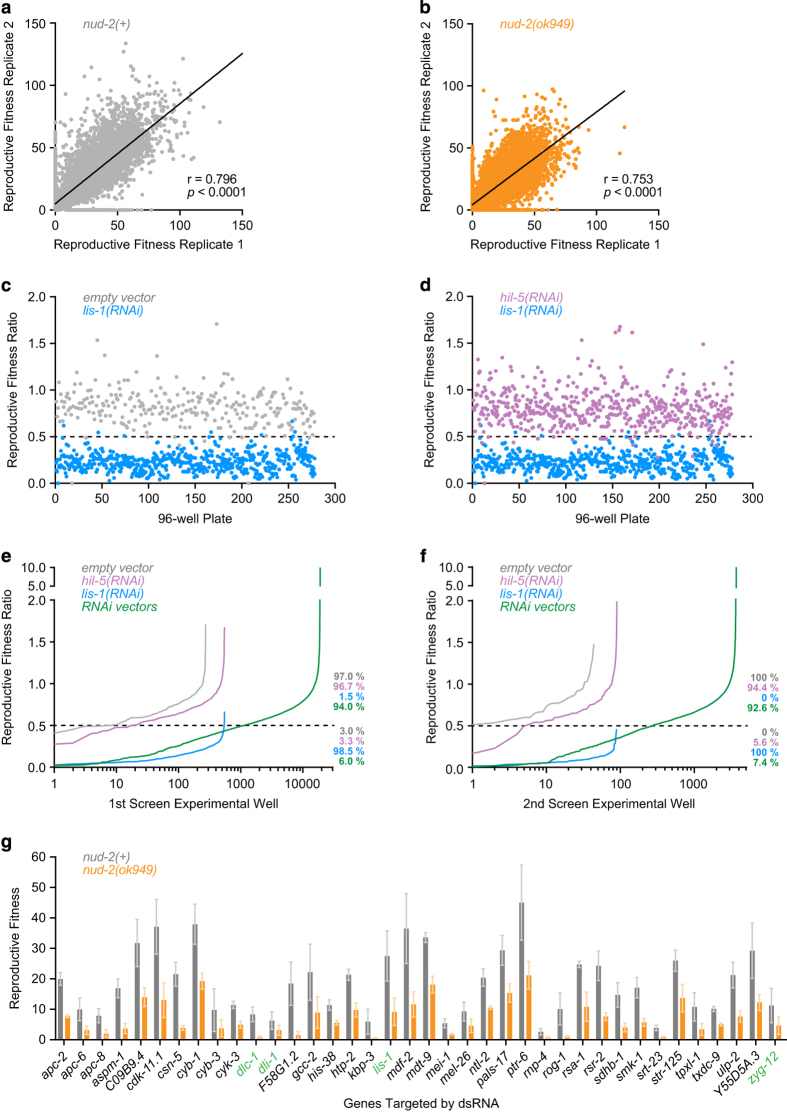
Results of the genome-wide RNAi screen. (**a**), (**b**) Correlation plots of the two technical replicates in the *nud-2(+)* strain *(a)* and the *nud-2(ok949)* strain *(b)* from the genome-wide primary screen. The Pearson correlation coefficient (r) and *p* value indicating statistical significance are indicated. (**c**), (**d**) Global comparison of positive and negative controls. For each 96-well plate, the reproductive fitness ratio is plotted for empty dsRNA vector versus *lis-1(RNAi) (c)* and *hil-5(RNAi)* versus *lis-1(RNAi) (d)*. The dashed line represents the cutoff used for hit selection. (**e**), (**f**) RF ratio distribution in the primary *(e)* and secondary *(f)* screen. Percentages indicate the fraction of negative controls (gray and magenta), positive controls (blue), and library dsRNA vectors (green) above and below the cutoff used for hit selection (dashed line). (**g**) Secondary screening results. Genes with an average RF ratio ≤ 0.5 from three biological replicates performed in duplicate were considered hits. RF is plotted for each gene in *nud-2(+)* and *nud-2(ok949)* with error bars representing the SEM. Genes in green font code for proteins known to directly associate with dynein.

An important ubiquitous dynein co-factor is the conserved nuclear distribution gene E (NudE), which was first discovered in filamentous fungi^6,7^. NudE and the related protein NudE-like (Nudel) directly bind dynein and the dynein co-factor Lis1. NudE/Nudel and Lis1 contribute to many cellular functions of dynein, and mutant NudE causes the brain developmental disease microcephaly^8,9^. On the molecular level, NudE/Nudel help tether Lis1 to dynein to regulate force production of the motor and mediate interactions between dynein and cargo^4,5^ (Fig. 1a). The nematode *Caenorhabditis elegans* possesses a single NudE/Nudel gene, *nud-2*, and animals in which *nud-2* is inhibited by RNAi-mediated knockdown or by the genetic deletion *nud-2(ok949)* display phenotypes consistent with compromised dynein function^10–12^. The *nud-2(ok949)* deletion removes 87 % of the open reading frame and is therefore likely a null allele^10^, yet viability and fertility of mutant animals is only mildly affected. Importantly, reducing LIS-1 levels in *nud-2(ok949)* embryos results in enhanced lethality relative to *nud-2(+)* controls (Fig. 1b, c), suggesting that *nud-2(ok949)* represents a sensitized background that can be exploited for the discovery of dynein pathway genes. We therefore implemented an RNAi-based genetic interactor screen by targeting 87 % of annotated *C. elegans* open reading frames in *nud-2(ok949)* and *nud-2(+)* control animals using the bacterial feeding library from the Ahringer group^13^, adapted to a 96-well liquid format (Fig. 1d). The primary screen consisted of two technical replicates per bacterial clone for a total of 19762 clones. Synchronized L1 larvae were allowed to grow and have progeny by feeding on the bacteria for 92 h. Animals were marked with green fluorescent protein (GFP) expressed in the pharynx (*myo-2::gfp*), which facilitated automated counting of parents and larval progeny on a fluorescence microscopy platform at the end of RNAi treatment (Fig. 1e, f). The number of progeny per parent, termed reproductive fitness, could then be calculated for each bacterial clone. Genes that when targeted with dsRNA decreased reproductive fitness in *nud-2(ok949)* animals at least two-fold relative to *nud-2(+)* animals were chosen for secondary screening (1077 clones) (Fig. 2a-e). Secondary screening was more stringent, consisting of 3 biological replicates, each carried out in 2 technical replicates (Fig. 2f). We present a final list of 38 genetic interactors (Fig. 2g; Table 1). The list includes 13 genes whose functions relate to the microtubule cytoskeleton, and 4 genes that code for known binding partners of dynein. Strikingly, 19 out of the 38 genes are implicated in embryonic cell division, where dynein is known to have multiple essential functions^14^. Further study of the *nud-2* interacting gene set promises to provide insight into the regulatory mechanisms that underlie dynein's functional diversity. Furthermore, re-analysis of the screening data using different cutoffs may extend the list of putative dynein pathway genes. Finally, the data on reproductive fitness in the control strain may serve as a useful reference for other genome-wide RNAi screens.

## Methods

### Worm strains

*C. elegans* strains were maintained at 16 °C or 20 °C on nematode growth media (NGM) plates seeded with OP50 bacteria. The following strains were used: *wild-type* (N2 Bristol isolate), GCP66 [*nud-2*(*ok949*)I 6 x outcrossed with N2], GCP68 [*nud-2*(*ok949*)I; *heEx526*(*myo-2p*::GFP)] from crossing GCP66 with SV1069^15^, and GCP95 [*heEx526*(*myo-2p*::GFP)] derived from the same cross but selected for the absence of *nud-2*(*ok949*).

### Worm growth and RNA interference

The genome-wide RNAi screen was performed using a modified version of the screening protocol in the 96-well liquid format developed by Lehner *et al.*^16^, and an automated screening platform was implemented based on the strategy previously reported by Maia *et al.*^15^ (Fig. 1d). Bacteria from the Ahringer library glycerol stock^17^ (Source BioScience) harboring expression vectors for gene-specific RNAi were transferred into 100 μL of LB medium (supplemented with 100 μg/mL ampicillin and 12.5 μg/mL tetracycline hydrochloride) in 96-well plates (#82.1582.001, Sarstedt) using a 96-pin replicator (Boekel) and grown overnight. The first column of pins was removed from the replicator to leave the first column of each plate free for inclusion of positive and negative controls. The negative controls were empty dsRNA vector pL4440 and *hil-5* dsRNA vector, which exhibited 10–20 % embryonic lethality in the *nud-2(ok949)* background. *plk-1* dsRNA vector, which causes 100 % embryonic lethality, was used as a control for RNAi efficiency, and *lis-1* dsRNA vector was used as a positive control for enhanced lethality in the *nud-2*(*ok949*) background (Fig. 1b, c). 5 μL of the saturated bacterial cultures were used to inoculate 400 μL of LB (with ampicillin and tetracycline) in a deep-well 96-well plate (#260251, Thermo Scientific Nunc), followed by incubation overnight. After induction of dsRNA expression with 4 mM IPTG for 1 h, bacteria were pelleted and re-suspended in 400 μL of complete S Medium using the Liquidator 96 Manual Pipetting System (Mettler Toledo). 10 μL of a synchronized L1 larval stage suspension, containing approximately 7 worms in 10 μL of M9 Buffer (Fig. 1e), were dispensed into each well of a flat-bottom 96-well plate (#260836, Thermo Scientific) using a Multidrop Combi Reagent Dispenser (Thermo Scientific), and 40 μL of bacterial suspension was added. Plates were incubated at 20 °C with shaking (200 rpm) for 92 h, which was sufficient time for the L1 larvae to grow until adulthood, lay eggs, and for these eggs to develop into larvae. Each 96-well library plate with bacteria was assayed in two technical replicates per strain.

### Image acquisition and automated counting of parents and progeny

Prior to image acquisition, 70 μL of worm paralyzing solution (50 μL complete S Medium, 20 μL of 0.7 % tricaine / 0.07 % tetramisole) was added to all wells. Brightfield and fluorescence images of each well were acquired with the IN Cell Analyzer 2000 (GE Healthcare) using a 2x / 0.1 NA Plan Fluor objective (Nikon). This allowed the entire well to be captured in a single image (Fig. 1f). Automated counting of adult worms (parents, larger pharynxes) and larvae (progeny, smaller pharynxes) was performed using the IN Cell Investigator Developer Toolbox software (GE Healthcare), and visual data inspection was performed with Spotfire DecisionSite (GE Healthcare) (Fig. 1f).

Automated counting involved two steps: 1) recognition of the pharynx by detection of objects with an area larger than 0.383 μm^2^, a mass (sum of all pixels within the shape) smaller than 300000 pixels, and a perimeter smaller than 1 mm; 2) distinction between larvae and adults by calculating the total intensity of pixels inside the object and applying a threshold. Only wells with 3-13 parent worms were considered for further analysis, as the number of progeny correlates linearly with the number of adults within this range (Fig. 1e).

### Data analysis and hit selection

ScreenSifter software was used to visualize and analyze the data^18^. Reproductive Fitness (RF) was defined as the number of progeny per parent^15^. The RF ratio for each dsRNA vector was calculated by dividing the RF of the *nud-2*(*ok949*) strain (GCP68) by the RF of the *nud-2(+)* control strain (GCP95) (Fig. 1b, c). After genome-wide screening with two technical replicates per strain (Data Record 1), a threshold for hit selection was defined based on the RF ratios for the positive control (*lis-1* dsRNA vector), which was included in each 96-well plate: $threshold={µ}_{p}+2\sigma_{p}$, where μ_p_ is the mean and σ_p_ is the standard deviation of the positive control RF ratios (Fig. 2c, d). The resulting threshold RF ratio was 0.43. dsRNA vectors that reduced viable progeny by more than 85 % in the *nud-2(+)* control strain were excluded, as the RF ratio could not be reliably calculated. Using these criteria, a total of 1077 dsRNA vectors with an RF ratio ≤ 0.5 were selected for secondary screening (Data record 2). dsRNA vectors with a mean RF ratio ≤ 0.5 from three biological replicates (2 technical replicates each) were included in a final list of genetic interactors (Fig. 2g; Table 1). All dsRNA vectors in the final hit list were sequenced with the M13 forward primer (5′ GTAAAACGACGGCCAGT 3′) to confirm the identity of the targeted gene (Table 1).

## Data Records

The data associated with this work is available as two downloadable spreadsheets from FigShare (Table 2; Data Citation 1).

### Data record 1 – primary screen

Raw data from the primary genome-wide RNAi screen is shown in five separate worksheets. The “all_data” worksheet shows the results for every bacterial clone. The worksheet “controls” contains the data for the positive and negative controls. The worksheet “undetermined” identifies the wells whose RF ratio could not be determined for technical reasons. The worksheet “lethal” lists genes that caused more than 85 % embryonic lethality in the *nud-2(+)* control strain and were therefore excluded from further analysis. The worksheet “hits” shows the dsRNA vectors selected for secondary screening and their location in the re-arrayed plates.

Column headers:

**Plate** - Ahringer library plate number.

**Well** - Well coordinates.

**WellType** - Content description. ‘S’, dsRNA vector; ‘CN1’, negative control - empty vector; ‘CN2’, negative control - *hil-5(RNAi)*; ‘CP’, positive control - *lis-1(RNAi)*; ‘C’, control for RNAi efficiency - *plk-1(RNAi)*; ‘E’, empty well.

**Reagent_ID** - Gene sequence identification as annotated in the Ahringer library database.

**Ad / Prg_R1 / R2** - Number of adults and progeny obtained in each technical replicate (R1, R2) for *nud-2(+)* in blue and *nud-2*(*ok949*) in red.

**RF_R1 / R2 / Avg** - Reproductive Fitness of each replicate and the mean of both. Occasionally the RF of a replicate could not be determined for technical reasons (marked “OUT”). In these cases, the value in RF_Avg corresponds to the RF value of the single replicate that worked.

**RF_ratio** - the RF ratio was obtained by dividing the RF_Avg of *nud-2(ok949)* by the RF_Avg of *nud-2(+)*.

**Gene Symbol** - Approved name of the gene targeted by the dsRNA vector in each bacterial clone.

### Data record 2 – secondary screen

Data obtained from the secondary RNAi screen is shown in three different worksheets. Analogous to Data Record 1, the worksheet “all_data” shows the results for every bacterial clone that was re-screened. The worksheet “controls” contains the data for the positive and negative controls. The worksheet “RF ratios” summarizes the RF ratios obtained for all clones in each biological replicate, and the average of all replicates. Genes that scored as “enhancers” are highlighted in green.

## Technical Validation

### Replicate correlation

Correlation analysis of replicates in the primary and secondary screen attests to the reproducibility of the screening procedure. In the primary screen, the Pearson correlation coefficients for the technical replicates were 0.80 for *nud-2(+)* and 0.75 for *nud-2*(*ok949*) (Fig. 2a, b). Pearson correlation coefficients for technical replicates of the three biological replicates in the secondary screen were 0.75, 0.74, and 0.71 for *nud-2(+)*, and 0.80, 0.77, 0.62 for *nud-2*(*ok949*). For pairwise correlations between the three biological replicates in the secondary screen, the Pearson correlation coefficients were 0.46 (R1, R2), 0.30 (R1, R3), and 0.47 (R2, R3) for *nud-2(+)*, and 0.46 (R1, R2), 0.42 (R1, R3) and 0.55 (R2, R3) for *nud-2(ok949)*. For all correlations, *p* < 0.0001.

### Controls

Controls were included in every 96-well plate, and plotting of their RF ratios demonstrates high consistency throughout the screening procedure with a clear separation between positive and negative controls (Fig. 2c, d). The robust performance of controls justifies the use of the positive control RF ratios as the basis for setting the threshold for hit identification (RF ratio ≤ 0.5; Fig. 2c, d).

### Genes in the final hit list with functional links to dynein

The final hit list is enriched in genes whose functions relate to the microtubule cytoskeleton (13 out of 38) and to cell division (19 out of 38), where dynein is known to play multiple essential roles^14^. In addition, we found two subunits of the dynein complex (*dli-1* and *dlc-1*) and two known dynein regulators (*lis-1* and *zyg-12*). This demonstrates that the screen was successful in identifying genes that functionally intersect with the dynein pathway.

## Usage Notes

The raw data of the two-stage RNAi screen is made accessible for several reasons (Data Records 1 and 2). First, it provides a list of genetic *nud-2* interactors, whose further study should advance our understanding of dynein regulation and function. Second, users can apply their own normalization strategies and thresholds to compare the *nud-2(+)* and *nud-2(ok949)* strains, which may extend the list of candidate dynein pathway genes. Third, while this study focused on genes whose inhibition reduces reproductive fitness of *nud-2*(*ok949*), some dsRNA vectors resulted in decreased lethality in *nud-2*(*ok949*) (RF ratio > 1; Fig. 2e, f). These genes are also potential interactors of *nud-2.* Fourth, the data on reproductive fitness in the control strain may serve as a useful reference for other genome-wide RNAi screens using the 96-well liquid format.

## Additional information

**How to cite this article**: Rocha, H. *et al.* A genome-scale RNAi screen for genetic interactors of the dynein co-factor *nud-2* in *Caenorhabditis elegans*. *Sci. Data* 5:180047 doi: 10.1038/sdata.2018.47 (2018).

**Publisher’s note**: Springer Nature remains neutral with regard to jurisdictional claims in published maps and institutional affiliations.

## Supplementary Material



## Figures and Tables

**Table 1:** genetic interactors of *nud-2(ok949)*.

Ahringer library coordinate	Sequence	Name	Description (human ortholog)
III-4C20	K06H7.6	*apc-2*	Subunit 2 of anaphase promoting complex (APC2)
II-6A23	F10B5.6	*apc-6*	Subunit 6 of anaphase promoting complex (APC6)
III-1K01	F10C5.1	*apc-8*	Subunit 8 of anaphase promoting complex (APC8)
I-4F17	C45G3.1	*aspm-1*	Abnormal Spindles homolog (ASPM)
IV-2J13	C09B9.4	C09B9.4	Tau tubulin kinase 2 (TTBK2)
II-5D02	B0495.2	*cdk-11.1*	Cyclin dependent kinase 11A (CDK11A)
IV-2N08	B0547.1	*csn-5*	Subunit 5 of COP9 signalosome complex (CSN5)
IV-9G19	ZC168.4	*cyb-1*	Cyclin B
V-10K05	T06E6.2	*cyb-3*	Cyclin B
III-3M05	ZK328.1	*cyk-3*	Ubiquitin C-terminal hydrolase
III-3O12	T26A5.9	*dlc-1*	Dynein light chain (DYNLL1 & DYNLL2)
IV-7A03^a^	C39E9.14	*dli-1*	Dynein light intermediate chain (DYNC1LI1 & DYNC1LI2)
II-10L01	F58G1.2	F58G1.2	Predicted nucleic acid binding activity
III-5A04	T05G5.9	*gcc-2* b	Golgin A1 (GOLGA1)
X-3F18^a^	K03A1.6	*his-38*	H4 histone
IV-10D16	Y73B6BL.2	*htp-2*	Meiosis-specific HORMA domain-containing protein
X-7N15^a^	F26H11.1	*kbp-3* b	Subunit of NDC80 complex (SPC25)
III-6L07	T03F6.5	*lis-1*	Lis1
IV-8J08	Y69A2AR.30	*mdf-2*	Mitotic arrest deficient 2 (MAD2)
IV-7I13	Y62E10A.11	*mdt-9*	RAB interacting factor (RABIF)
I-4G05	T01G9.5	*mei-1*	Catalytic subunit of katanin
I-4D17	ZK858.4	*mel-26*	Substrate adaptor of CUL-3-containing E3 ubiquitin ligase
II-3F06	B0286.4	*ntl-2*	CCR4-NOT transcription complex subunit 2 (CNOT2)
III-6P24	Y82E9BR.13	*pals-17*	Protein containing ALS2cr12 (ALS2CR12) domain
II-4E16	C54A12.1	*ptr-6*	Sterol sensing domain protein (PTCHD3)
III-2A02^a^	R07E5.14	*rnp-4*	Putative member of exon-exon junction complex
II-11G09	F54D12.6	*rog-1*	Ras activating factor (FRS2/FRS3)
I-5I05^a^	C25A1.9	*rsa-1*	PP2A regulatory subunit of the B'' class
II-11F20	Y57A10A.19	*rsr-2*	SRRM2 (serine/arginine repetitive matrix 2 & 3)
II-6N13	F42A8.2	*sdhb-1*	Succinate dehydrogenase subunit B (SDHB)
V-6K13	F41E6.4	*smk-1*	Dictyostelium suppressor of MEK null homolog (SMEK)
IV-1C20^a^	Y55F3C.2	*srt-23*	Serpentine Receptor, class T
V-14G09	T05E12.1	*str-125*	Seven transmembrane receptor
I-7L01	Y39G10AR.12	*tpxl-1*	Targeting Protein for Xenopus Klp2-like (TPX2)
III-3K22	C05D11.3	*txdc-9*	Thioredoxin domain-containing protein (TXNDC9)
II-4K17	Y38A8.3	*ulp-2*	SUMO specific peptidase (CD180, SENP6, TLR4)
III-7G20	Y55D5A.3	Y55D5A.3	N-acylethanolamine acid amidase (NAAA)
II-4I13	ZK546.1	*zyg-12*	Hook protein

^a^Clones with multiple predicted RNAi targets (WormBase v. WS257).

^b^Sequenced gene was different from expected RNAi target.

**Table 2:** Data records.

Source	Protocol	Samples	Data
GCP95	Feeding RNAi	TechnicalRep1	Data Record 1
GCP95	Feeding RNAi	TechnicalRep2	Data Record 1
GCP68	Feeding RNAi	TechnicalRep1	Data Record 1
GCP68	Feeding RNAi	TechnicalRep2	Data Record 1
GCP95	Feeding RNAi	BiologicalRep1, TechnicalRep1	Data Record 2
GCP95	Feeding RNAi	BiologicalRep1, TechnicalRep2	Data Record 2
GCP95	Feeding RNAi	BiologicalRep2, TechnicalRep1	Data Record 2
GCP95	Feeding RNAi	BiologicalRep2, TechnicalRep2	Data Record 2
GCP95	Feeding RNAi	BiologicalRep3, TechnicalRep1	Data Record 2
GCP95	Feeding RNAi	BiologicalRep3, TechnicalRep2	Data Record 2
GCP68	Feeding RNAi	BiologicalRep1, TechnicalRep1	Data Record 2
GCP68	Feeding RNAi	BiologicalRep1, TechnicalRep2	Data Record 2
GCP68	Feeding RNAi	BiologicalRep2, TechnicalRep1	Data Record 2
GCP68	Feeding RNAi	BiologicalRep2, TechnicalRep2	Data Record 2
GCP68	Feeding RNAi	BiologicalRep3, TechnicalRep1	Data Record 2
GCP68	Feeding RNAi	BiologicalRep3, TechnicalRep2	Data Record 2
